# The effect of stand-alone and additional preoperative video education on patients’ knowledge of anaesthesia: A randomised controlled trial

**DOI:** 10.1097/EJA.0000000000002109

**Published:** 2024-12-19

**Authors:** Sander F. van den Heuvel, Philip Jonker, Sanne E. Hoeks, Sohal Y. Ismail, Robert Jan Stolker, Jan-Wiebe H. Korstanje

**Affiliations:** From the Department of Anaesthesiology, Erasmus MC University Medical Centre Rotterdam, CA Rotterdam, Netherlands (SFvdH, PJ, SEH, RJS, JWHK), the Department of Psychiatry, Erasmus MC University Medical Centre Rotterdam, Rotterdam, The Netherlands (SYI)

## Abstract

**BACKGROUND:**

Fully digital preoperative information could save valuable time and resources. However, compared with face to face consultations, equivalent levels of safety, patient satisfaction and participation need to be maintained when using other methods to inform patients. This trial compared knowledge retention between preoperative stand-alone video education and face-to-face education by an anaesthesiologist.

**OBJECTIVES:**

To assess if video education, alone or combined with face-to-face education, leads to better knowledge retention more than conventional face-to-face education.

**DESIGN:**

A randomised controlled trial with four arms: Video, Anaesthesiologist, Video & Anaesthesiologist, and Reference for baseline measurements and exploration of a test-enhanced learning effect.

**SETTING:**

A Dutch tertiary care centre from February 2022 to February 2023.

**PATIENTS:**

A total of 767 adult patients undergoing preoperative consultation for elective non-cardiothoracic surgery, with 677 included in the complete case analysis.

**INTERVENTION(S):**

Stand-alone preoperative video education and video education in combination with face-to-face education in the preoperative outpatient clinic.

**MAIN OUTCOME MEASURES:**

Primary outcome, measured by the Rotterdam Anaesthesia Knowledge Questionnaire, was knowledge retention on day 0. Secondary outcomes included knowledge retention at 14 and 42 days, preoperative anxiety, and the need for additional information using the Amsterdam Preoperative Anxiety and Information Scale. Other outcomes were satisfaction, self-assessed knowledge, and test-enhanced learning effect.

**RESULTS:**

Stand-alone video education led to higher Rotterdam Anaesthesia Knowledge Questionnaire scores than face-to-face education on day 0: median [IQR], 87.5 [81.3 to 93.8] vs. 81.3 [68.8 to 87.5], *P* < 0.001. Combined education in the “Video & Anaesthesiologist” group led to better knowledge retention compared with both the “Anaesthesiologist” group and the Video group: 93.8 [87.5 to 93.8] vs. 81.3 [68.8 to 87.5], *P* < 0.001; 93.8 [87.5 to 93.8] vs. 87.5 [81.3 to 93.8], *P* = 0.01, respectively. No differences in the patients’ preoperative anxiety and satisfaction levels were found.

**CONCLUSION:**

Compared with face-to-face education by an anaesthesiologist, stand-alone video and combined video education improve short-term knowledge retention, without increasing patient anxiety.

**TRIAL REGISTRATION:**

ClinicalTrials.gov Identifier: NCT05188547.

## Graphical abstract

**Figure:**
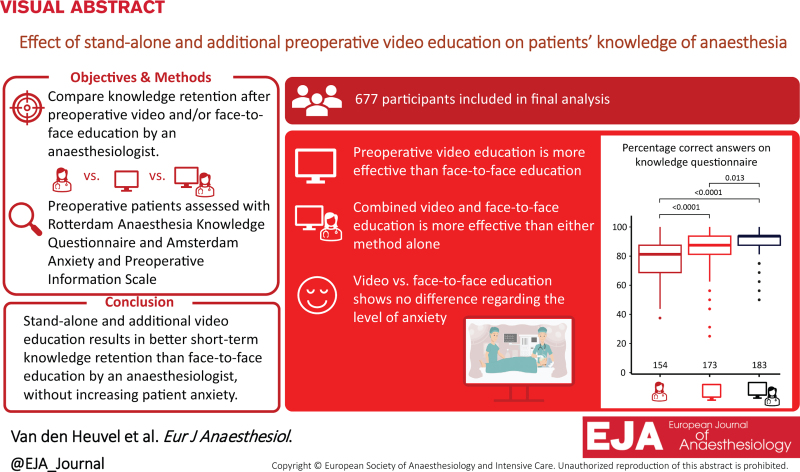

**KEY POINTS**

- Preoperative video education is more effective than face-to-face education.
- Combined video and face-to-face education is more effective than either of the methods alone.
- Video vs. face-to-face education shows no difference regarding levels of patient anxiety.
- Satisfaction and objective knowledge level show only a weak correlation.
- Subjective and objective knowledge level show only a weak correlation.

## Introduction

Preoperative patient education about anaesthesia and obtaining informed consent is an ethical obligation and legal requirement and is therefore an integral part of anaesthetic consultations. Face-to-face consultations are the predominant practice in most countries, driven by legal requirements pertaining to the informed consent procedure. However, the effectiveness and efficiency of this face-to-face approach may vary, particularly for patients who are in good health or undergoing minor surgical procedures.^1–9^ A transition to fully digital consultations could save valuable time and resources in the preoperative process.^4,10,11^ However, compared with face-to-face consultations, perioperative safety, patient satisfaction and patient participation need to be maintained when using other methods to inform patients.

Video education is a potential supplement to face-to-face consultations, improving patient understanding and reducing anxiety.^12–16^ However, in anaesthesiology, trials did not explore stand-alone video education and focused only on short-term knowledge retention.^17–19^ Furthermore, the questionnaires used in these trials were not validated and control groups were mostly absent. This limits definitive conclusions for further digitisation of the preoperative anaesthetic processes.

Therefore, we conducted a randomised controlled trial (RCT) to compare short-term (day 0) knowledge retention in adult patients undergoing preoperative anaesthesiological screening for elective non-cardiothoracic surgery. We used three different educational methods: stand-alone video education, face-to-face education with an anaesthesiologist and a combination of both. Secondary objectives were to compare mid-term (day 14) and long-term (day 42) knowledge retention, and the level of preoperative anxiety and need for information on day 0 between the three educational groups and with the reference group.

## Material and methods

The trial was conducted at the preoperative screening clinic of the Department of Anaesthesiology of the Erasmus MC University Medical Centre, Rotterdam, the Netherlands. It was registered before patient enrolment on ClinicalTrials.gov (NCT05188547, https://clinicaltrials.gov/ct2/show/NCT05188547, principal investigator: J.-W.H. Korstanje, registration date: 12 January 2022) and followed the CONSORT standard for multi-arm parallel group RCTs.^20^ This trial (Ethical Committee No. MEC-2021-0769) was deemed not to be subject to Dutch Law on Medical Research by the Medical Ethics Committee of Erasmus MC, Rotterdam, the Netherlands (chairperson Prof. H.W. Tilanus) on 11 November 2021. It adhered to the Declaration of Helsinki and written informed consent for the study was obtained from all participants included in the analysis. During the preparation of this work the authors used ChatGPT 4.0 translate and refine the language and clarity of content. The authors reviewed and edited the content as needed and take full responsibility for the content of the publication.

### Trial design and patient allocation

This was a single-centre, randomised, four-arm trial at Erasmus MC, Rotterdam. In the Netherlands all patients have their anaesthetic consultations well in advance of the scheduled surgery, sometimes days, often weeks or even months. Patients presenting at the preoperative outpatient clinic for this anaesthetic consultation and meeting the inclusion criteria (aged 18 or over, scheduled for elective non-cardiothoracic surgery) were considered for participation from February 2022 to February 2023. The exclusion criteria were the inability to understand or read and write in Dutch. After oral consent for the study, patients were digitally registered and randomly allocated to one of the four groups using trial software developed by NovaCair B.V.: “Anaesthesiologist”, “Video”, “Video & Anaesthesiologist” or “Reference”. The latter served to provide a reference level for secondary outcomes and assess a potential enhanced-learning effect of doing a test on their knowledge before receiving information.^21^ Written (digital) consent for the study was required before viewing the videos or completion of the questionnaires. Anaesthesiologists educating the participants were blinded to participant allocation. Both patients and anaesthesiologists were blinded to the results.

Patients were allowed to have a family member or any person the patient relies on present while watching the video, filling out the questionnaires, and preoperative face-to-face consultations. While this was not actively encouraged, it reflects common practice. Patients were instructed not to look up information on the internet to ensure the integrity of their responses.

### Procedures

#### Face-to-face consultation

Dutch law requires a patient to have a face-to-face consultation with an anaesthesiologist before consent for anaesthesia is obtained. Therefore, all participants, including ‘Video’ group participants, received standard care, that is, face-to-face anaesthesiologist consultation on day 0. However, the timing of this consultation differed based on the trial arm to which the participant was allocated: the video group watched the videos and received their first knowledge test before seeing the anaesthesiologist. Anaesthesiologists were free to determine how to educate their patients provided they complied with national guidelines, reflecting the current standard of care. This required discussing all applicable anaesthesia techniques, including general anaesthesia. Informed consent for anaesthesia was obtained and documented in the patient's file during this consultation.

#### Video education

The participants allocated to the ‘Video’ and ‘Video & Anaesthesiologist’ group were asked to watch two animated videos, one on general anaesthesia and one about preoperative instructions.^22^ The videos were designed by NovaCair in collaboration with Behandeling Begrepen (translation: Treatment Understood) B.V. (Amsterdam, the Netherlands). The two videos were less than 8 min long and were seen only at the preoperative clinic on day 0. The participants watched the videos alone, or with their ‘family member’ in a dedicated room in the clinic.

### Data collection and outcome assessment

#### Data collection

Pseudonymised questionnaire results were processed via an ISO 27001 certified cloud service by NovaCair B.V. Baseline characteristics were extracted from the hospital's electronic system and stored securely. Participants reported their highest education level and surgical history. The first questionnaire was completed at the preoperative visit. ‘Anaesthesiologist’, ‘Video’ and ‘Video & Anaesthesiologist’ groups were invited by email to complete the knowledge questionnaire on days 14 and 42, with reminders sent for nonresponses. The ‘Reference’ group was not assessed on these days.

#### Assessment of primary outcome: knowledge retention

The primary outcome was short-term (day 0) knowledge retention in patients undergoing preoperative screening for elective non-cardiothoracic surgery, measured using the Rotterdam Anaesthesia Knowledge Questionnaire (RAKQ). This was assessed after one of three educational methods: video education, face-to-face education, or a combination of both. The RAKQ, a psychometrically validated Dutch tool (Table 1, Supplemental Digital Content), was used to objectively measure the level of knowledge.^23^ It covered topics on day-of-surgery expectations (GA-I), anaesthesia side-effects and complications (GA-II), and generic information and instructions (GEN) (Table 1, Supplemental Digital Content). In this study, all parts of the Rotterdam Anaesthesia Knowledge Questionnaire (RAKQ) are weighted equally, with each question contributing the same weight to the final score. We calculated the total score by summing all correct answers and expressing this as a percentage of the total number of questions. This approach ensures a straightforward and easily interpretable assessment of participants’ knowledge. While the final stages of the original questionnaire development incorporated Item Response Theory (IRT) models, we opted to use only the validated questions from the initial development steps. This choice allows for a simpler scoring system, which avoids the added complexity of the IRT model, ensuring clarity and consistency in how participant knowledge is evaluated.

Secondary outcomes were mid-term (day 14) and long-term (day 42) knowledge retention after these educational methods. These intervals align with our hospital's preoperative anaesthesiological screening to surgery timeline: one-third of patients have surgery within 14 days of the preoperative consultation, another third within 42 days, and the rest after 42 days. The ‘Video’ group, despite the initial standalone video education, also had the mandatory face-to-face consultation. We hypothesised that the ‘Video’ group would have an advantage over the ‘Video & Anaesthesiologist’ group due to a test-enhanced learning effect as a result of completing the RAKQ before the face-to-face consultation with the anaesthesiologist on day 0. In simple terms, testing aids retention of knowledge, identifies gaps in that knowledge, and participants learn more from the next learning episode. To study this testing effect, the Reference group participants did not see the videos, but completed the RAKQ before their face-to-face consultation with an anaesthesiologist.

#### Assessment of preoperative anxiety and need for information

**Fig. 1:**
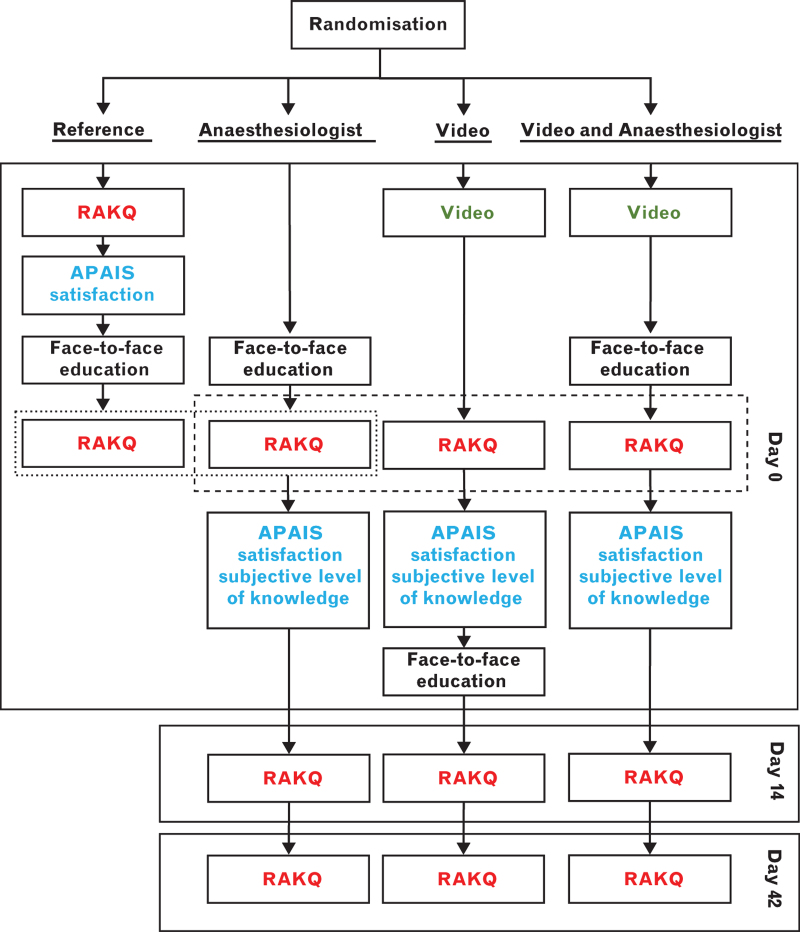
Trial design.

Other secondary outcomes were anxiety and the need for information, measured using the Dutch version of the Amsterdam Preoperative Anxiety and Information Scale (APAIS,^24^ Table 2, Supplemental Digital Content). These were assessed after the three educational methods, but before and after education in the ‘Reference’ group (Fig. 1). The ‘Reference’ group results served as a baseline for comparisons. Anxiety was scored on a 4–20 scale (APAIS items 1, 2, 4 and 5), and need for information on a 2–10 scale (APAIS items 3 and 6), with higher scores indicating higher levels of anxiety or need for information.

Assessment and procedures regarding: knowledge retention (in red) and preoperative anxiety, need for information, satisfaction and subjective level of knowledge (in blue). Dashed line, primary outcome measurement; Dotted line, test-enhanced learning measurement). APAIS, Amsterdam Preoperative Anxiety and Information Scale; RAKQ, Rotterdam Anaesthesia Knowledge Questionnaire.

#### Assessment of additional outcome parameters

Satisfaction with the provided information was measured using a five-point Likert scale question after each educational method and before and after education in the ‘Reference’ group. Subjective knowledge was evaluated by self-assessment using three questions on a five-point Likert scale (Table 2, Supplemental Digital Content).

At 14 and 42 days, when knowledge was measured again, the ‘Video’ group may benefit from a test-enhance learning effect. On day 0, this group completed the knowledge questionnaire after the video but before face-to-face education, while the ‘Video & Anaesthesiologist’ group did so after both. We measured the knowledge level in the ‘Reference’ group twice to explore this effect (Fig. 1), comparing them with the ‘Anaesthesiologist’ group, which was assessed once after face-to-face education.

### Sample size

An unpublished pilot trial was conducted before this current study to assess the feasibility and determine an effect size for sample size calculation. The effect size (Cohen's *d*) between video and face-to-face education on day 14 knowledge retention was 0.46. A two-tailed *t*-test with an alpha of 0.017, corrected for multiple comparisons according to Bonferroni (0.05/3), a beta of 0.2, and a 33% expected loss to follow-up rate were used, resulting in a calculated sample size of 148 participants per trial arm.

### Statistical analysis

In our study we used a modified intention-to-treat analysis, including all participants who were randomised to the allocated intervention and gave written informed consent for the study.^25^ Because of missing primary outcome data (knowledge retention on day 0), a complete case analysis was performed.^26,27^ Data analysis was performed using R (version 4.2.2).^28^ Normality was assessed visually and with the Shapiro–Wilk test. The primary and secondary outcome parameters were expressed as median [IQR] scores and compared using the median differences (95% CI) and the Mann–Whitney *U* test, with *P* values corrected for multiple comparisons, with two-sided *P* value ≤0.05 after correction considered to be significant.^29^ Additional analyses explored satisfaction, subjective knowledge levels, and the test-enhanced learning effect. Correlations were calculated using Spearman rank correlation coefficients and presented as rho (95% CI). For further analysis of the relation between satisfaction, subjective level of knowledge and objective level of knowledge, these measures were divided into two groups: higher and lower levels of the parameter. Satisfaction and subjective knowledge were split into ‘Low’ (those neutral or strongly disagreeing with at least one) and ‘High’ (those strongly agreeing with all statements). Objective knowledge was split in a group with <80%, and a group with ≥80% correct answers on the RAKQ. Lost to follow-up rates were also presented, and changes in correct answer percentages on the RAKQ at different time points were described for participants who completed the full trial.

## Results

**Fig. 2:**
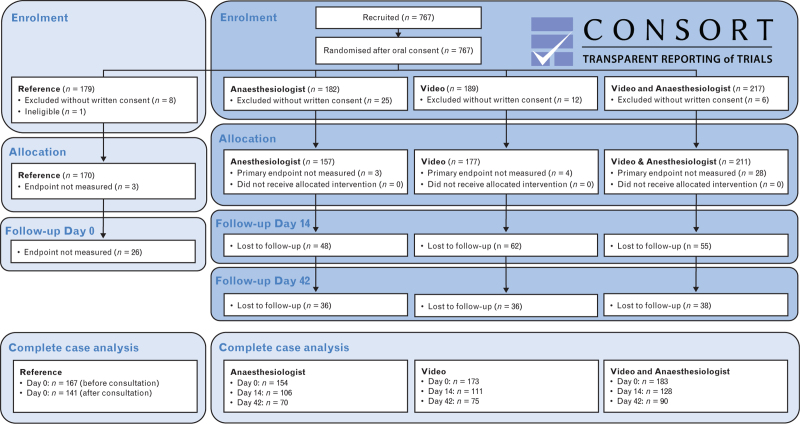
CONSORT flow diagram for multi-arm parallel group randomised controlled trials.

In this trial, 767 participants were recruited and randomised (Fig. 2). Baseline characteristics of the 715 participants who provided written informed consent for the study are presented in Table 1, and those of the 677 participants with no missing outcome measurements, as included in the complete case analysis, are presented in Table 3, Supplemental Digital Content. Both analysis groups were balanced, except the ‘Video’ group had more locoregional and less general anaesthesia counselling. The median [IQR] age was 55.0 [41.0 to 65.0] years, and 344 (48.1%) participants were female. Five hundred seventy-five (80.4%) participants reported previous surgery, 311 (43.5%) participants had completed tertiary education, 197 (27.6%) participants were ASA-PS 3 or 4, and 588 (82.2%) participants were counselled by the anaesthesiologist for general anaesthesia as the primary anaesthesia technique.

**Table 1:** Baseline characteristics

	Reference	Anaesthesiologist	Video	Video & Anaesthesiologist	SMD^a^
	*n* = 170	*n* = 157	*n* = 177	*n* = 211	
Age; years	56.5 [41.0 to 66.0]	54.0 [42.0 to 64.0]	56.0 [42.0 to 68.0]	53.0 [39.5 to 64.0]	0.066
Sex; female	83 (48.8)	81 (51.6)	76 (42.9)	104 (49.3)	0.088
Prior surgery					0.132
Yes	141 (82.9)	128 (81.5)	151 (85.3)	155 (73.5)	
No	25 (14.7)	22 (14.0)	22 (12.4)	24 (11.4)	
Unknown	4 (2.4)	4 (2.5)	0 (0)	4 (1.9)	
Missing	0 (0)	3 (1.9)	4 (2.3)	28 (13.3)	
Highest level of education					0.156
Primary	3 (1.8)	5 (3.2)	7 (4.0)	2 (0.9)	
Secondary	86 (50.6)	80 (51.0)	84 (47.5)	90 (42.7)	
Tertiary	77 (45.3)	68 (43.3)	80 (45.2)	86 (40.8)	
Unknown	4 (2.4)	1 (0.6)	2 (1.1)	5 (2.4)	
Missing	0 (0)	3 (1.9)	4 (2.3)	28 (13.3)	
ASA-PS					0.117
1	25 (14.7)	20 (12.7)	28 (15.8)	40 (19.0)	
2	95 (55.9)	95 (60.5)	99 (55.9)	116 (55.0)	
3	47 (27.6)	39 (24.8)	48 (27.1)	53 (25.1)	
4	3 (1.8)	3 (1.9)	2 (1.1)	2 (0.9)	
Anaesthesia technique					0.241
General anaesthesia	139 (81.8)	133 (84.7)	140 (79.1)	176 (83.4)	
Locoregional anaesthesia	2 (1.2)	6 (3.8)	13 (7.3)	5 (2.4)	
Spinal anaesthesia	2 (1.2)	4 (2.5)	7 (4.0)	3 (1.4)	
PSA	27 (15.9)	14 (8.9)	17 (9.6)	27 (12.8)	
Specialism					^b^
Dermatology	6 (3.5)	4 (2.5)	3 (1.7)	6 (2.8)	
ENT	19 (11.2)	24 (15.3)	19 (10.7)	24 (11.4)	
Gastroenterology	9 (5.3)	4 (2.5)	1 (0.6)	15 (7.1)	
General surgery	47 (27.6)	44 (28.0)	43 (24.3)	55 (26.1)	
Gynaecology	12 (7.1)	8 (5.1)	4 (2.3)	12 (5.7)	
Maxillofacial surgery	2 (1.2)	3 (1.9)	6 (3.4)	9 (4.3)	
Neurosurgery	12 (7.1)	12 (7.6)	9 (5.1)	18 (8.5)	
Ophthalmology	4 (2.4)	4 (2.5)	1 (0.6)	7 (3.3)	
Orthopaedic surgery	12 (7.1)	9 (5.7)	22 (12.4)	11 (5.2)	
Pain medicine	3 (1.8)	4 (2.5)	3 (1.7)	2 (0.9)	
Plastic surgery	11 (6.5)	19 (12.1)	25 (14.1)	14 (6.6)	
Pulmonology	5 (2.9)	0 (0)	4 (2.3)	3 (1.4)	
Radiology	3 (1.8)	1 (0.6)	3 (1.7)	5 (2.4)	
Radiotherapy	2 (1.2)	2 (1.3)	2 (1.1)	0 (0)	
Traumatology	4 (2.4)	6 (3.8)	6 (3.4)	6 (2.8)	
Urology	19 (11.2)	13 (8.3)	26 (14.7)	24 (11.4)	

Values are median [IQR] or number (%). ASA-PS, American Society of Anaesthesiologists − Physical Status; PSA, procedural sedation and analgesia; SMD, standardised mean difference.

a The pooled standardised mean difference calculated across all pairwise group comparisons.

b Standardised mean differences (SMD) were not calculated for individual surgical specialties due to the low number of participants in each category.

### Knowledge retention

**Fig. 3:**
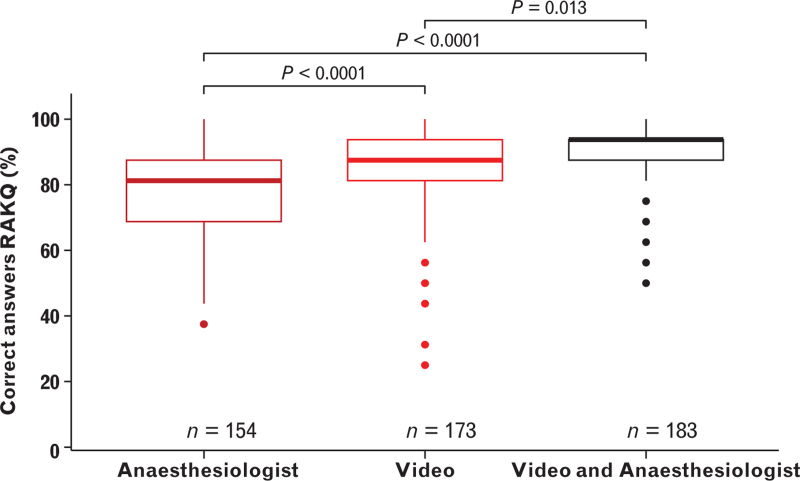
Percentage of correct answers on the RAKQ on day 0 in the Anesthesiologist, Video and “Video & Anesthesiologist” groups.

The primary outcome is presented in Fig. 3 as the median percentage of correct answers on the RAKQ on day 0. The ‘Video’ group had a higher median [IQR] percentage of correct RAKQ answers (87.5 [81.3 to 93.8]%) than the ‘Anaesthesiologist’ group (81.3 [68.8 to 87.5]%), with a median difference (95% CI) of 6.3 (6.2 to 12.5)%, *P* < 0.001. The ‘Video & Anaesthesiologist’ group scored higher (93.8 [87.5 to 93.8]%) than both the ‘Anaesthesiologist’ group (median difference (95% CI) 12.5 (6.3 to 12.5)%, *P* < 0.001) and the ‘Video’ group (median difference (95% CI) 0.0 (0.0 to 6.2)%, *P* = 0.01).

*P* values per comparison of two groups.

At day 14, as a secondary outcome for mid-term knowledge retention (day 14), the ‘Video’ group had a significantly higher median [IQR] percentage of correct RAKQ answers (93.8 [81.3 to 93.8]%) than the ‘Anaesthesiologist’ group (87.5 [75.0 to 93.8]%), with a median difference (95% CI) of 6.2 (0.0 to 6.3)%, *P* = 0.003. The ‘Video & Anaesthesiologist’ group (93.8 [87.5 to 100]%) scored significantly higher than both the ‘Anaesthesiologist’ group (median difference (95% CI) 6.3 (6.2 to 12.5)%, *P* < 0.001) and the ‘Video’ group (median difference (95% CI) 0.0 (0.0 to 6.2)%, *P* = 0.02).

At day 42, the ‘Video & Anaesthesiologist’ group scored higher (93.8 [87.5 to 100]%) than the ‘Anaesthesiologist’ group (87.5 [75.0 to 93.8]%), with a median difference (95% CI) of 6.2 (0.0 to 6.3)%. Other comparisons showed no difference. See Table 2 for RAKQ results at all time points.

**Table 2:** Percentage of correct answers after education administered by an Anaesthesiologist, by Video or a combination of Video & Anaesthesiologist

	Anaesthesiologist		Video		Video & Anaesthesiologist		*P*-values^a^		
	Correct	*n*	Correct	*n*	Correct	*n*	A vs. V	A vs. VA	V vs. VA
Day 0	81.3 [68.8 to 87.5]	154	87.5 [81.3 to 93.8]	173	93.8 [87.5 to 93.8]	183	<0.001	<0.001	0.01
Day 14	87.5 [75.0 to 93.8]	106	93.8 [81.3 to 93.8]	111	93.8 [87.5 to 100]	128	<0.001	<0.001	0.02
Day 42	87.5 [81.3 to 93.8]	70	93.8 [87.5 to 100]	75	93.8 [87.5 to 100]	90	0.07	0.002	0.23

Percentage of correct values are presented as median [IQR]. A, Anaesthesiologist; V, Video; VA, Video & Anaesthesiologist.

a Corrected for multiple comparisons using the Holm method.

### Preoperative anxiety and need for information

The secondary outcome on preoperative anxiety and need for information is presented in Table 3. No differences were found in the scores on both subscales between the ‘Anaesthesiologist’, ‘Video’ and ‘Video & Anaesthesiologist’ groups (*P* > 0.99 for all comparisons). However, all groups did show improvements on both subscales when they were compared with the baseline reference, as measured before education in the ‘Reference’ group. The improvements on the ‘Preoperative anxiety’ subscale were statistically significant in all three educational groups vs. baseline reference (median differences (95% CI), −1.0 (−2.0 to 0.0), *P* = 0.02). Figure 1, Supplemental Digital Content presents the results on the individual items of the APAIS for each trial group.

**Table 3:** Preoperative anxiety, need for information and satisfaction

	Reference^d^	Anaesthesiologist	Video	Video & Anaesthesiologist
	*n* = 167	*n* = 154	*n* = 173	*n* = 183
APAIS				
Need for information^a^	7.0 [5.5 to 8.0]	6.0 [5.0 to 8.0]	6.0 [5.0 to 8.0]	6.0 [5.0 to 8.0]
Preoperative anxiety^b^	9.0 [6.0 to 11.5]	7.0 [5.0 to 10.0]	7.0 [6.0 to 10.0]	7.0 [5.0 to 10.0]
Satisfied with information^c^	3.0 [2.5 to 4.0]	5.0 [4.0 to 5.0]	5.0 [4.0 to 5.0]	5.0 [5.0 to 5.0]

Values are presented as median [IQR]. APAIS, Amsterdam Preoperative Anxiety and Information Scale.

a Range from 2 to 10.

b Range from 4 to 20.

c Range from 1 to 5.

d Scores obtained before education.

### Additional results

#### Satisfaction

The ‘Video’ group showed no significant difference in the score for satisfaction compared with the ‘Anaesthesiologist’ group (*P* = 0.39), as shown in Table 3. The ‘Video & Anaesthesiologist’ group showed a significantly higher median [IQR] score of 5.0 [5.0 to 5.0] on satisfaction compared with both the “Anaesthesiologist” group, 5.0 [4.0 to 5.0], median difference (95% CI) of 0.0 (0.0 to 0.0), *P* < 0.001; and the ‘Video’ group 5.0 [4.0 to 5.0], with a median difference (95% CI) of 0.0 (0.0 to 0.0), *P* = 0.002.

Spearman rank correlation coefficient for satisfaction and objective level of knowledge was 0.14 (95% CI 0.06, 0.23, *P* = 0.001). In Table 4, Supplemental Digital Content, the participants’ satisfaction with the provided information, dichotomised, is presented in relation to dichotomised scores on the RAKQ. It demonstrates that 116 (23.4%) participants in the ‘High’ group regarding satisfaction, scored < 80% on the RAKQ. Figure 2, Supplemental Digital Content presents a more detailed figure on the relation between satisfaction and the objective knowledge level, with different cut-off values of the RAKQ.

#### Subjective knowledge level

Spearman rank correlation coefficient for the subjective level of knowledge and objective level of knowledge was 0.11 (95% CI 0.02, 0.19, *P* = 0.013). Table 4, Supplemental Digital Content presents the dichotomised subjective knowledge levels of the participants in relation to dichotomised scores on the RAKQ. It shows that 79 (19.4%) participants in the ‘High’ group regarding the subjective knowledge level, scored <80% correct answers on the RAKQ, whereas 59 (57.3%) participants in the ‘Low’ group regarding the subjective knowledge level scored ≥80% correct answers on the RAKQ. Supplemental Digital Content 6 (figure) presents a more detailed figure on the relation between the subjective and objective knowledge level, with different cut-off values.

#### Test-enhanced learning

The group without the pre-test, i.e. the ‘Anaesthesiologist’ group, showed no difference in the median [IQR] percentage of correct answers on day 0 (81.3 [68.8 to 87.5]%), compared with the group with the pre-test, i.e. the ‘Reference’ group on day 0, second measurement (81.3 [75.0 to 87.5]%) with a median difference (95% CI) of 0.0 (−6.3 to 0.0)%, *P* = 0.07).

#### Lost to follow-up

Of all participants in the complete case analysis (*n* = 677), 165 (32.4%) were lost to follow-up by day 14 and an additional 110 (31.9%) by day 42, resulting in 402 participants who completed the full trial period. The median [IQR] age of those who were lost to follow-up was 51.0 [37.0 to 63.0] years, compared with 57.0 [49.0 to 67.0] years for those who completed the trial. The proportion of males was higher among those who were lost to follow-up (57.8%) than among those who completed the trial (44.7%).

#### Knowledge retention over time

Table 5, Supplemental Digital Content shows the median [IQR] percentage of correct answers on the RAKQ over time on days 0, 14 and 42, within each group, only of the participants who completed the full trial.

## Discussion and conclusion

This RCT compared knowledge retention after video and face-to-face education by an anaesthesiologist. Stand-alone video education led to better short-term (day 0) knowledge retention than face-to-face education. Combining both methods improved short-term retention further. These advantages persisted in the mid-term (day 14). In the long-term (day 42), only patients having the combination of video and face-to-face education retained more knowledge than face-to-face education. No difference was observed in preoperative anxiety or the need for additional information between the methods. These results align with systematic reviews by Tom *et al.* and Lee *et al.*, showing improved knowledge retention with video education supplementing face-to-face education.^16,17^

Additional analyses suggest satisfaction and perceived knowledge levels do not necessarily correspond to the objective knowledge level. About 20% of the participants who agreed with statements confirming adequate satisfaction or knowledge, scored <80% on the RAKQ. This implies that in a clinical context, when utilising questionnaires to assess subjective knowledge or satisfaction, patients with objective knowledge gaps may be overlooked although they may benefit from additional education.

No relevant learning enhancement was suggested by offering the RAKQ before face-to-face education by an anaesthesiologist. Therefore, the timing of procedures and assessments should not have resulted in an advantage for the ‘Video’ group over the ‘Video & Anaesthesiologist’ group on days 14 and 42. No differences were detected within the groups in the percentage of correct answers on days 0, 14 and 42 within groups.

Video education, by design, took place only once and on the same day as face-to-face education. However, video education could provide a more adaptable approach for patients, allowing them to watch and re-watch videos at their convenience. This would further accentuate the advantage of video education, whether used as stand-alone or in conjunction with face-to-face education.

Implementing video education requires access to digital devices and internet connectivity. These technological barriers could particularly affect older or less tech-savvy patients, potentially limiting the effectiveness of video education in these populations. Addressing these barriers is crucial for the successful integration of digital educational tools in clinical practice.

### Limitations

This study has several limitations. To begin with, participants could not be blinded to the received educational method, which might have influenced their responses, thereby potentially introducing performance bias. However, the assessors were blinded, and the assessments were standardised to minimise the impact of a potential performance bias.

Additionally, participants were randomised after verbal consent for the study. However, some participants, particularly in the ‘Anaesthesiologist’ group, did not complete the digital study form, including the written consent. This introduced selection bias, as we could not include these participants in the analysis despite their initial verbal agreement. To assess the potential impact of this selection bias, we calculated the standardised mean differences (SMD) for all baseline characteristics. We found only a slight imbalance regarding the primary anaesthesia technique choice, but no further imbalance was detected. To assess the impact on the primary outcome, we calculated the SMD between general anaesthesia and all other anaesthesia techniques. The means were 81.3 [68.8 to 87.5] for general anaesthesia and 84.3 [60.9 to 87.5] for other techniques, resulting in an SMD of 0.141, suggesting a minimal impact.

Furthermore, although we anticipated participant loss before days 14 and 42 measurements and we factored this into our initial sample size calculation, an attrition bias could not be prevented. Despite reminder emails to limit loss to follow-up, the rates remained high. However, the statistical inference on day 14 has sufficient power due to our a priori sample size determination. It is important to note that the loss to follow-up introduced a bias—participants lost were younger and more often male, implying that the results on days 14 and 42 are less generalisable than on day 0.

Another point to consider is the legal requirement of a face-to-face consultation with an anaesthesiologist as this led to the convergence of the ‘Video’ and ‘Video & Anaesthesiologist’ groups on days 14 and 42. Therefore, no definitive conclusions could be drawn regarding the effect of stand-alone video education on mid- and long-term knowledge retention.

In addition, given our study design, it was not feasible to determine in advance which patients would choose or receive general anaesthesia, as participants in the Video group did not see an anaesthesiologist before watching the videos. Therefore, we also included patients scheduled for regional anaesthesia or sedation. Limiting inclusion to surgery requiring general anaesthesia would have restricted the generalisability of our findings. In the Netherlands, general anaesthesia is always presented as an alternative, ensuring all patients were informed during their preoperative consultation. However, we acknowledge this may introduce variability in the informed consent process, potentially affecting generalisability. Furthermore, participants in the Video group, who completed the APAIS before consulting with an anaesthesiologist and had a strong preference for regional anaesthesia, might feel confused or experience increased anxiety when presented with information on general anaesthesia. This is despite being informed that the video education in this trial focused solely on general anaesthesia.

Lastly, we aimed to compare video education with real-life face-to-face consultation rather than using a scripted face-to-face consultation. This approach allows us to evaluate the effectiveness of different educational methods in a realistic clinical setting, providing more generalisable and practical insights. While this might also introduce variability in the content discussed, it better reflects actual clinical practice and patient experiences.

## Conclusion

Our study concludes that stand-alone video education outperforms face-to-face education by an anaesthesiologist in terms of preoperative knowledge retention. Additionally, combining video with face-to-face education is more effective than either method alone. To the best of our knowledge, this is the first study to show the superiority of stand-alone video education over traditional face-to-face consultation. This supports the potential for video education to ensure adequate patient knowledge and facilitate a transition to fully digital anaesthetic consultations. We propose integrating video education into standard practice to enhance mandatory face-to-face consultations.

## Supplementary Material

Supplemental Digital Content

## Supplementary Material

Supplemental Digital Content
