# Supplementary material for: The effect of stand-alone and additional preoperative video education on patients’ knowledge of anaesthesia: A randomised controlled trial
Source: Eur J Anaesthesiol. 2024 Dec 19;42(4):313–23. doi: 10.1097/EJA.0000000000002109 (PMC11872255; doi:10.1097/EJA.0000000000002109)
Supplement: Supplemental Digital Content [file ejanet-42-313-s001.docx]

# Supplemental Digital Content - Table 1.

# Rotterdam Anaesthesia Knowledge Questionnaire

|  | |
| --- | --- |
|  |  |
| ***Generic items*** | |
| 1 | Patients are generally seen in advance of the surgery by the same anaesthesiologist who administers the anaesthesia on the day of the surgery. |
|  | 1. *True* 2. *False* 3. *I do not know* |
| 2 | What can anaesthesiologists do to reduce anxiety? |
|  | 1. *Give patients a tablet to calm them* 2. *Put the patient under anaesthesia on the ward* 3. *Reschedule the operation for another day* 4. *I do not know* |
| 3 | What must you do with your usual daily medications? |
|  | 1. *The anaesthesiologist will discuss and arrange this with you. Usually, you may continue using your all medication* 2. *Don’t use any medication, bring all medication with you to the hospital to take it after the operation* 3. *You must consult your own general practitioner about this* 4. *I do not know* |
| 4 | Which of the fluids stated below may you drink up to 2 hours before the operation? |
|  | 1. *Freshly squeezed orange juice* 2. *Milk* 3. *Tea* 4. *I do not know* |
|  | |
| ***General anesthesia - I*** | |
| 1 | What do you notice from the breathing tube placed in your mouth during the operation? |
|  | 1. *It is often painful because the tube is placed before the start of general anaesthesia* 2. *Very little, maybe a brief period of throat pain and hoarseness after the surgery* 3. *This is placed under local anaesthesia before the start of general anaesthesia* 4. *I do not know* |
| 2 | How does an anaesthesiologist administer the anaesthesia drugs when putting a person to sleep? |
|  | 1. *By giving you a pill* 2. *By giving you spinal anaesthesia (an injection in the back)* 3. *By giving you an injection in a vein through a line.* 4. *I do not know* |
| 3 | Where do most patients wake up after general anaesthesia? |
|  | 1. *In the operating room, recovery room or intensive care unit* 2. *On the surgical ward where the patient was before surgery* 3. *At home* 4. *I do not know* |
| 4 | Who administers general anaesthesia? |
|  | 1. *A nurse* 2. *An anaesthesiologist* 3. *A surgeon* 4. *I do not know* |
| 5 | To insert the breathing tube, the anaesthesiologist makes a small incision in the wind pipe and reseals the opening at the end of the operation. |
|  | 1. *True* 2. *False* 3. *I do not know* |
| 6 | When is the breathing tube placed? |
|  | 1. *Before the patient goes to sleep* 2. *After the patient goes to sleep and before the operation begins* 3. *After the patient goes to sleep and the after operation has started* 4. *I do not know* |
|  | |
| ***General anaesthesia - II*** | |
| 1 | Under general anaesthesia, it is possible to develop a damaged nerve from being in a particular position for too long. |
|  | 1. *True* 2. *False* 3. *I do not know* |
| 2 | Sometimes, your teeth can be damaged during the placement of the breathing tube. |
|  | 1. *True* 2. *False* 3. *I do not know* |
| 3 | Patients must tell the anaesthesiologist if they have loose teeth. |
|  | 1. *True* 2. *False* 3. *I do not know* |
| 4 | After an operation under general anaesthesia, a patient’s ability to concentrate can be reduced for a short period of time. |
|  | 1. *True* 2. *False* 3. *I do not know* |
| 5 | Does every patient experience nausea after general anaesthesia? |
|  | 1. *No, only patients vulnerable to nausea* 2. *Yes, it is unavoidable* 3. *No, this rarely happens* 4. *I do not know* |
| 6 | Can general anaesthesia cause dementia? |
|  | 1. *Yes, it is unavoidable* 2. *No, this rarely happens* 3. *I do not know* |

**Supplemental Digital Content - Table 2. - Document
Questions on anxiety, subjective level of knowledge and satisfaction**

**The Amsterdam Preoperative Anxiety and Information Score (Moerman, van Dam et al. 1996):**

1. I am worried about the anaesthetic. Likert scale: Not at all – Extremely (1-5)
2. The anaesthetic is on my mind constantly. Likert scale: Not at all – Extremely (1-5)
3. I would like to know as much as possible about the anaesthetic. Likert scale: Not at all – Extremely (1-5)
4. I am worried about the procedure. Likert scale: Not at all – Extremely (1-5)
5. The procedure is on my mind constantly. Likert scale: Not at all – Extremely (1-5)
6. I would like to know as much as possible about the procedure. Likert scale: Not at all – Extremely (1-5)

**Subjective level of knowledge:**

1. I have a clear image of what to expect on the day of the general anaesthesia. Likert scale: No clear image – Very clear image (1-5)
2. The risks of general anaesthesia are clear to me. Likert scale: Not clear at all – Very clear (1-5)
3. I know what to do in preparation for the general anaesthesia. Likert scale: I don’t know that at all – I know that very well (1-5)

**Satisfaction:**

1. I am satisfied with the information I was given about the general anaesthesia. Likert scale: Not satisfied at all – Very satisfied (1-5)

**Supplemental Digital Content – Table 3**

**Baseline characteristics participants in complete case analysis**

*Table 1. Baseline characteristics participants in complete case analysis*

|  | **Reference** | **Anaesthesiologist** | **Video** | **Video & Anaesthesiologist** | **SMD*** |
| --- | --- | --- | --- | --- | --- |
|  | n=167 | n=154 | n=173 | n=183 |  |
| Age; years | 57.0 [40.5 to 66.0] | 53.5 [42.0 to 64.0] | 56.0 [42.0 to 67.0] | 53.0 [40.0 to 64.0] | 0.051 |
| Sex; female | 81 (48.5) | 80 (51.9) | 75 (43.4) | 91 (49.7) | 0.090 |
| Previous surgery; yes | 138 (82.6) | 128 (83.1) | 151 (87.3) | 155 (84.7) | 0.135 |
| **Highest level of education** |  |  |  |  | 0.157 |
| Primary | 3 (1.8) | 5 (3.2) | 7 (4.0) | 2 (1.1) |  |
| Secondary | 85 (50.9) | 80 (51.9) | 84 (48.6) | 90 (49.2) |  |
| Tertiary | 75 (44.9) | 68 (44.2) | 80 (46.2) | 86 (47.0) |  |
| Unknown | 4 (2.4) | 1 (0.6) | 2 (1.2) | 5 (2.7) |  |
| **ASA-PS** |  |  |  |  | 0.134 |
| 1 | 24 (14.4) | 19 (12.3) | 28 (16.2) | 34 (18.6) |  |
| 2 | 93 (55.7) | 94 (61.0) | 97 (56.1) | 101 (55.2) |  |
| 3 | 47 (28.1) | 38 (24.7) | 46 (26.6) | 47 (25.7) |  |
| 4 | 3 (1.8) | 3 (1.9) | 2 (1.2) | 1 (0.5) |  |
| **Anaesthesia technique** |  |  |  |  | 0.251 |
| General anaesthesia | 136 (81.4) | 130 (84.4) | 137 (79.2) | 154 (84.2) |  |
| Locoregional anaesthesia | 2 (1.2) | 6 (3.9) | 13 (7.5) | 3 (1.6) |  |
| Spinal anaesthesia | 2 (1.2) | 4 (2.6) | 7 (4.0) | 3 (1.6) |  |
| PSA | 27 (16.2) | 14 (9.1) | 16 (9.2) | 23 (12.6) |  |
| **Specialty** |  |  |  |  | 0.543 |
| Dermatology | 6 (3.6) | 3 (1.7) | 4 (2.6) | 6 (3.3) |  |
| ENT | 19 (11.4) | 18 (10.4) | 24 (15.6) | 20 (10.9) |  |
| Gastroenterology | 9 (5.4) | 1 (0.6) | 4 (2.6) | 13 (7.1) |  |
| General Surgery | 46 (27.5) | 42 (24.3) | 42 (27.3) | 51 (27.9) |  |
| Gynaecology | 12 (7.2) | 4 (2.3) | 8 (5.2) | 12 (6.6) |  |
| Maxillofacial Surgery | 2 (1.2) | 6 (3.5) | 3 (1.9) | 7 (3.8) |  |
| Neurosurgery | 12 (7.2) | 8 (4.6) | 11 (7.1) | 16 (8.7) |  |
| Ophthalmology | 4 (2.4) | 1 (0.6) | 4 (2.6) | 7 (3.8) |  |
| Orthopaedic Surgery | 12 (7.2) | 22 (12.7) | 9 (5.8) | 8 (4.4) |  |
| Pain Medicine | 3 (1.8) | 3 (1.7) | 4 (2.6) | 2 (1.1) |  |
| Plastic Surgery | 10 (6.0) | 24 (13.9) | 19 (12.3) | 12 (6.6) |  |
| Pulmonology | 5 (3.0) | 4 (2.3) | 0 (0) | 3 (1.6) |  |
| Radiology | 3 (1.8) | 3 (1.7) | 1 (0.6) | 3 (1.6) |  |
| Radiotherapy | 2 (1.2) | 2 (1.2) | 2 (1.3) | 0 (0) |  |
| Traumatology | 3 (1.8) | 6 (3.5) | 6 (3.9) | 5 (2.7) |  |
| Urology | 19 (11.4) | 26 (15.0) | 13 (8.4) | 18 (9.8) |  |

Values are median [IQR] or number (%). * The Pooled Standardised Mean Difference calculated across all pairwise group comparisons. ASA-PS, American Society of Anesthesiologists - Physical Status; PSA, Procedural sedation and analgesia; SMD, Standardised Mean Difference

**Supplemental Digital Content – Table 4**
**Subjective knowledge and Satisfaction vs Objective knowledge (RAKQ)**

|  | **Subjective knowledge level^1^** | | **Satisfaction^2^** | |
| --- | --- | --- | --- | --- |
|  | **Low** | **High** | **Low** | **High** |
| Percentage correct answers on the RAKQ | n=103 | n=407 | n=14 | n=496 |
| < 80% | 44 (42.7) | 79 (19.4) | 7 (50.0) | 116 (23.4) |
| ≥ 80% | 59 (57.3) | 328 (80.6) | 7 (50.0) | 380 (76.6) |

Values are number (%). RAKQ, Rotterdam Anaesthesia Knowledge Questionnaire. ^1^ Lowest Likert scale <4 or ≥ 4; ^2^  Lowest

Likert scale <4 or ≥ 4

**Supplemental Digital Content – Table 5**

**Percentage of correct answers over time after education administered by an Anaesthesiologist or by Video or a**

**combination of both.**

|  | **Day 0** | **Day 14** | **Day 42** | **P *values*** | | |
| --- | --- | --- | --- | --- | --- | --- |
|  | **% Correct** | **% Correct** | **% Correct** | **Day 0 vs 14** | **Day 14 vs 42** | **Day 0 vs 42** |
| Anaesthesiologist (n=70) | 87.5 [75.0 to 92.2] | 87.5 [81.3 to 93.8] | 87.5 [81.3 to 93.8] | 0.42 | 0.42 | 0.07 |
| Video (n=75) | 93.8 [87.5 to 100] | 93.8 [87.5 to 93.8] | 93.8 [87.5 to 100] | >0.99 | >0.99 | >0.99 |
| Video & Anaesthesiologist (n=90) | 93.8 [87.5 to 100] | 93.8 [87.5 to 100] | 93.8 [87.5 to 100] | 0.96 | 0.96 | 0.85 |

Sub-group analysis of the participants that completed the full trial. Number correct values are presented as median [IQR]. * Corrected for multiple comparisons using the Holm method.

| **Supplemental Digital Content - Figure 1**  **Preoperative Anxiety and Need for Information.** |
| --- |
|  |
| **Preoperative Anxiety** |
| **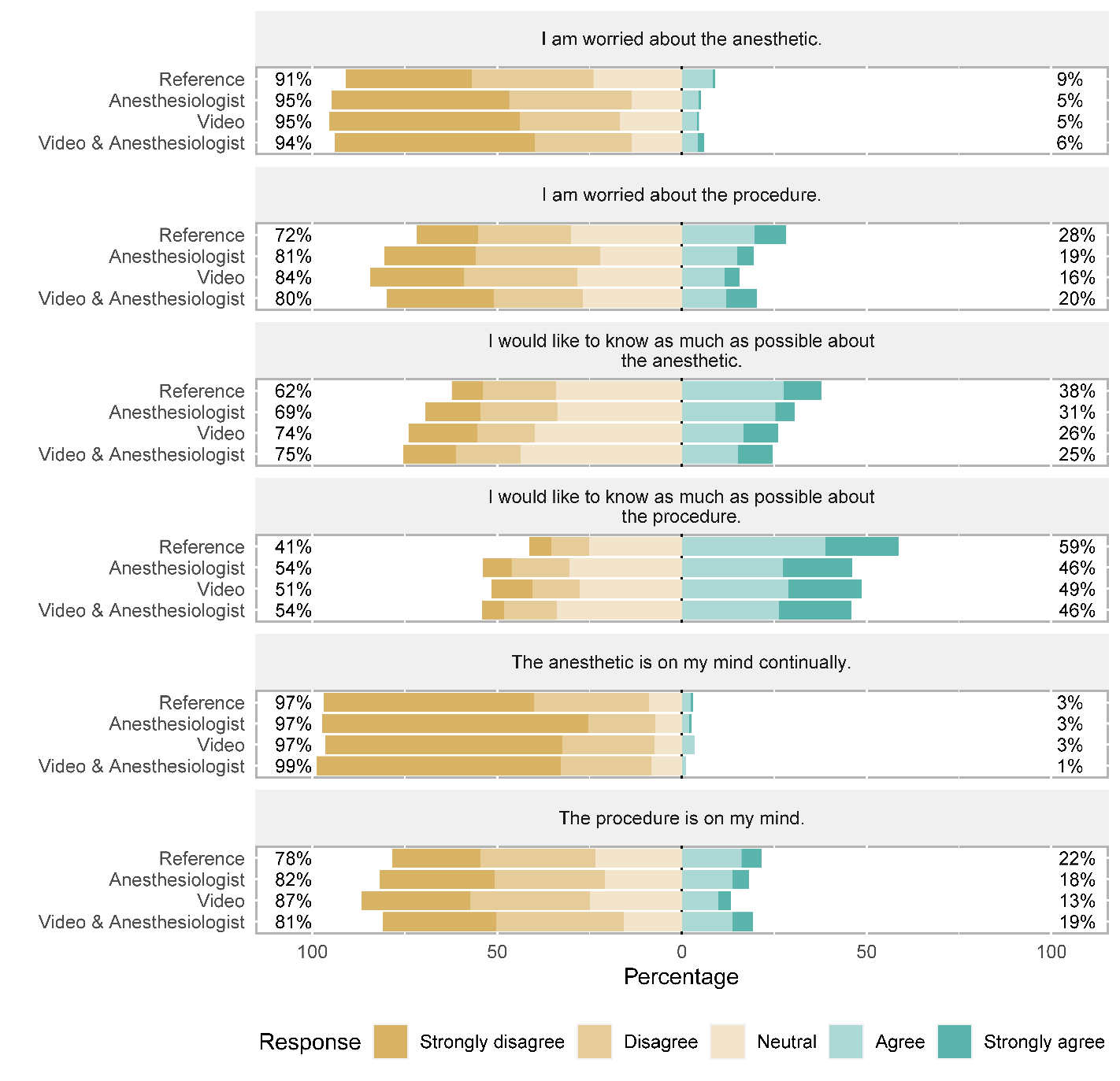**  **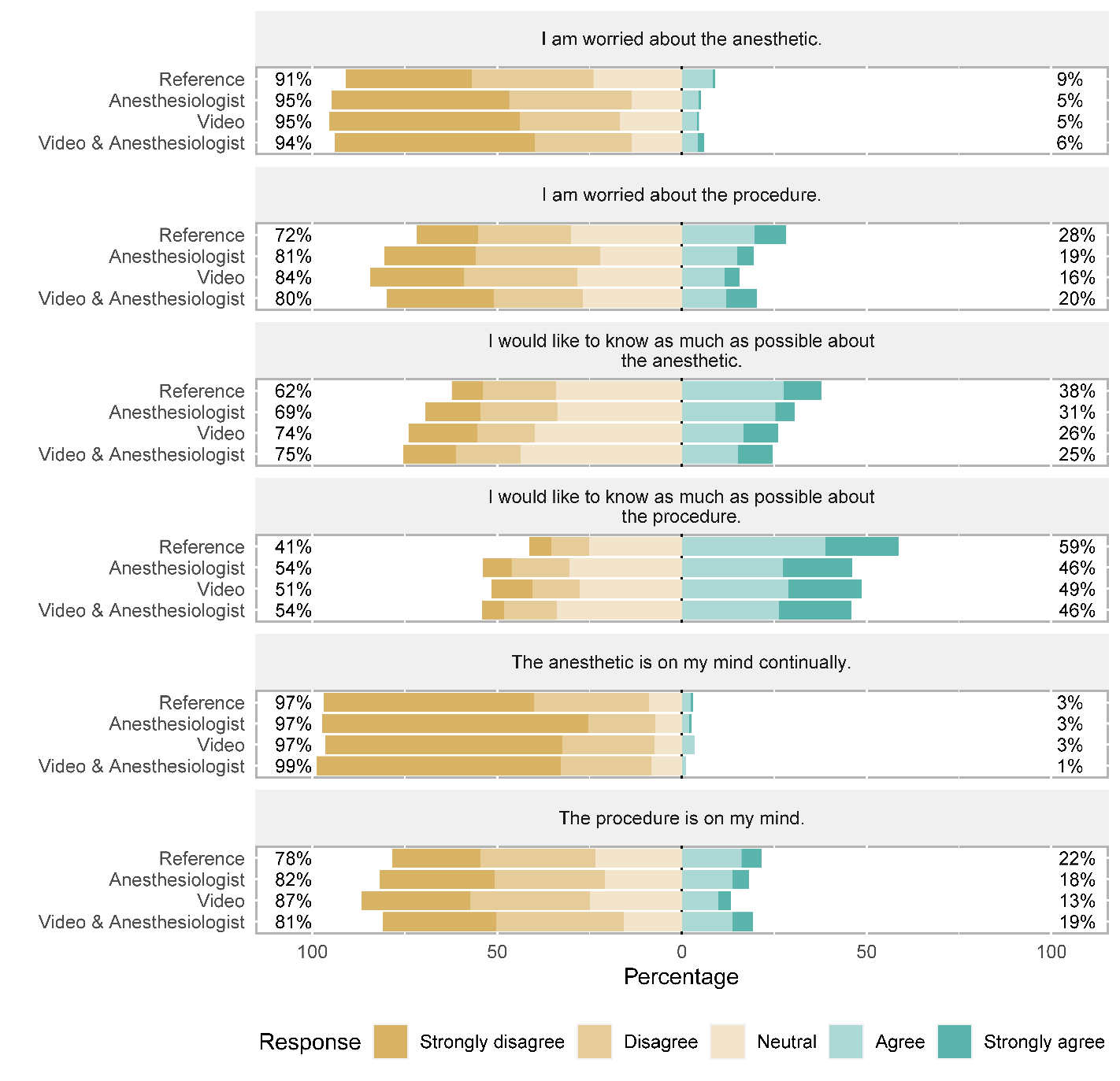** |
|  |
| **Need for Information** |
| **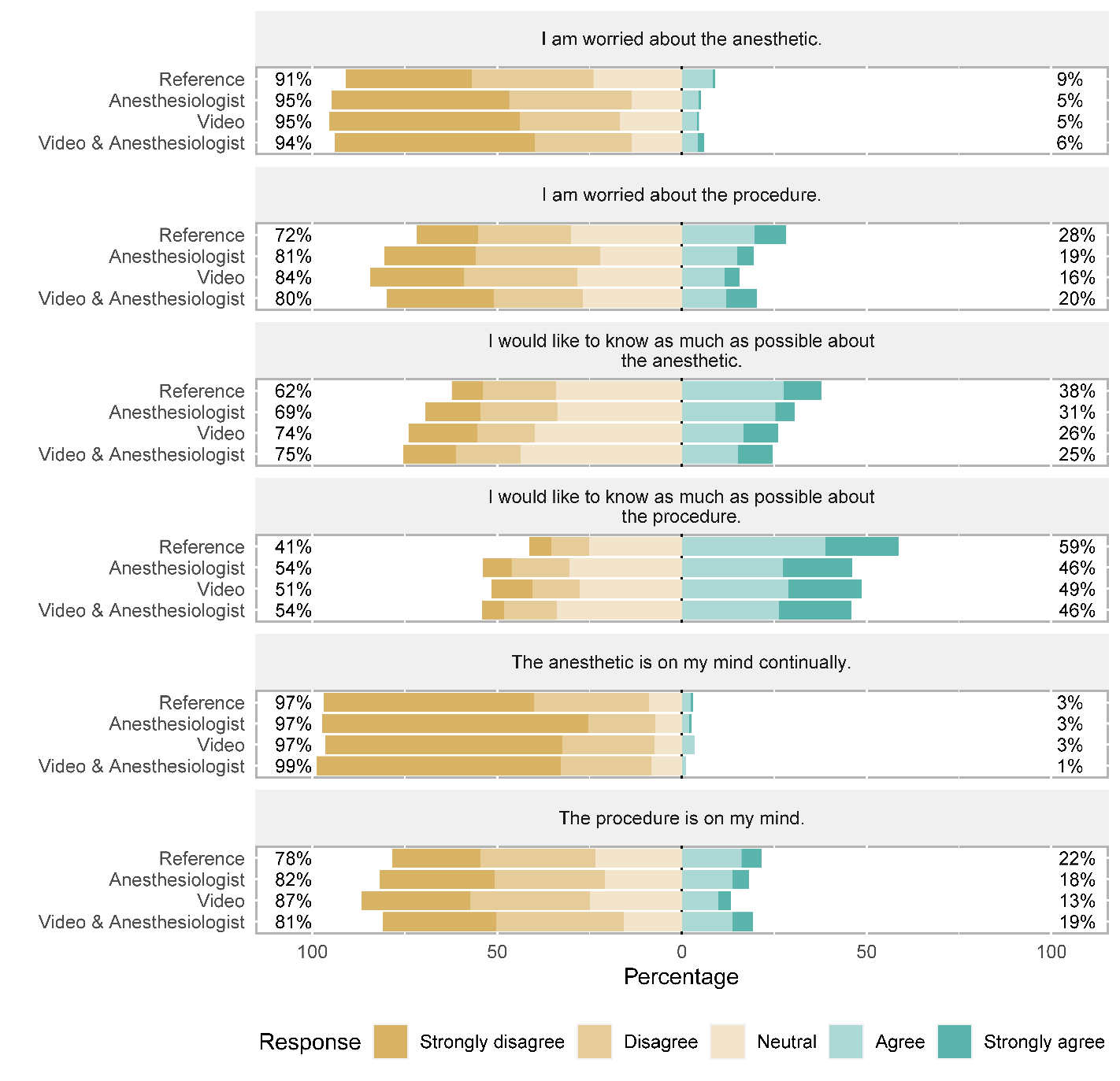**  **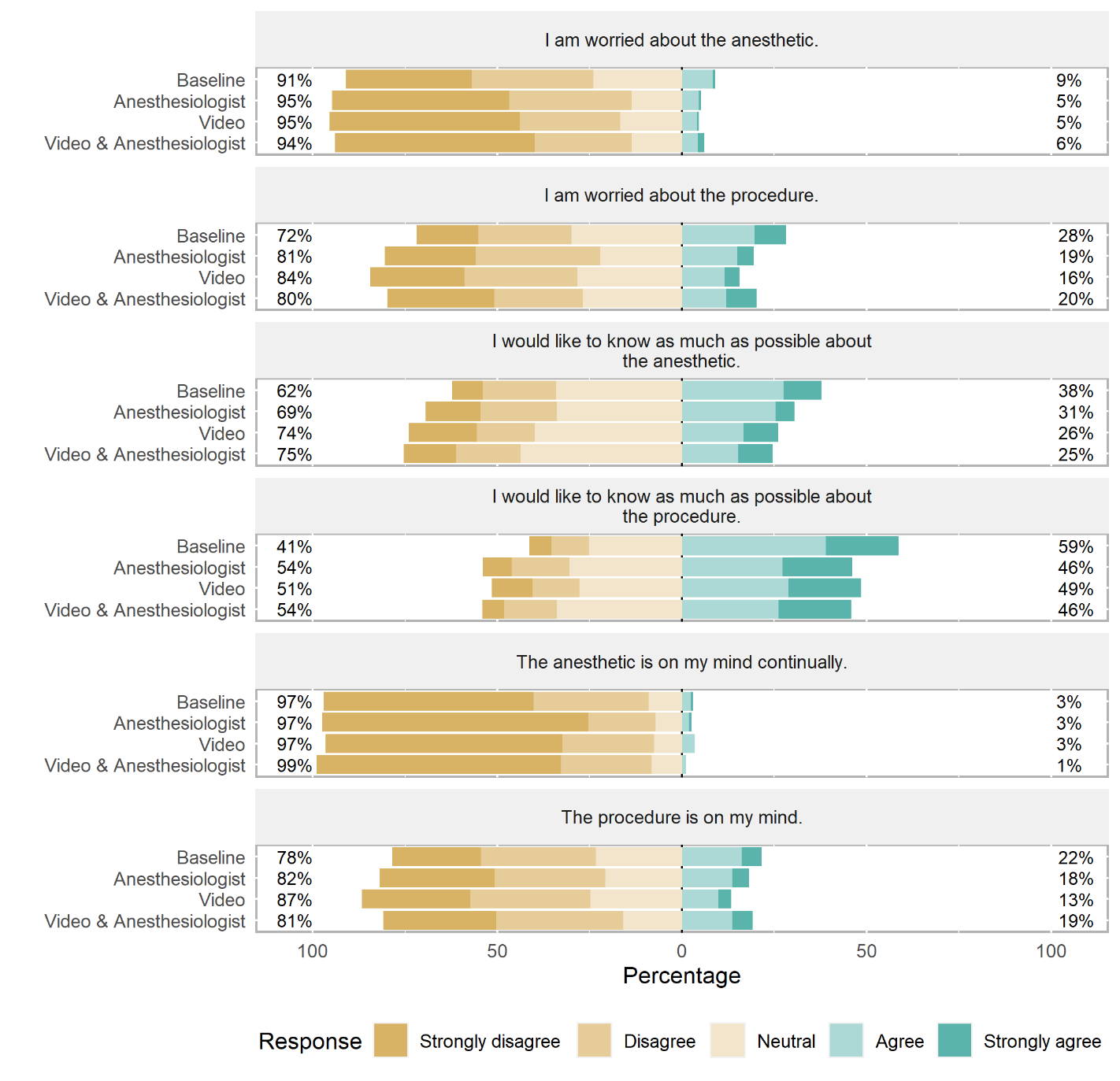** |
|  |
| **Distribution** |
| 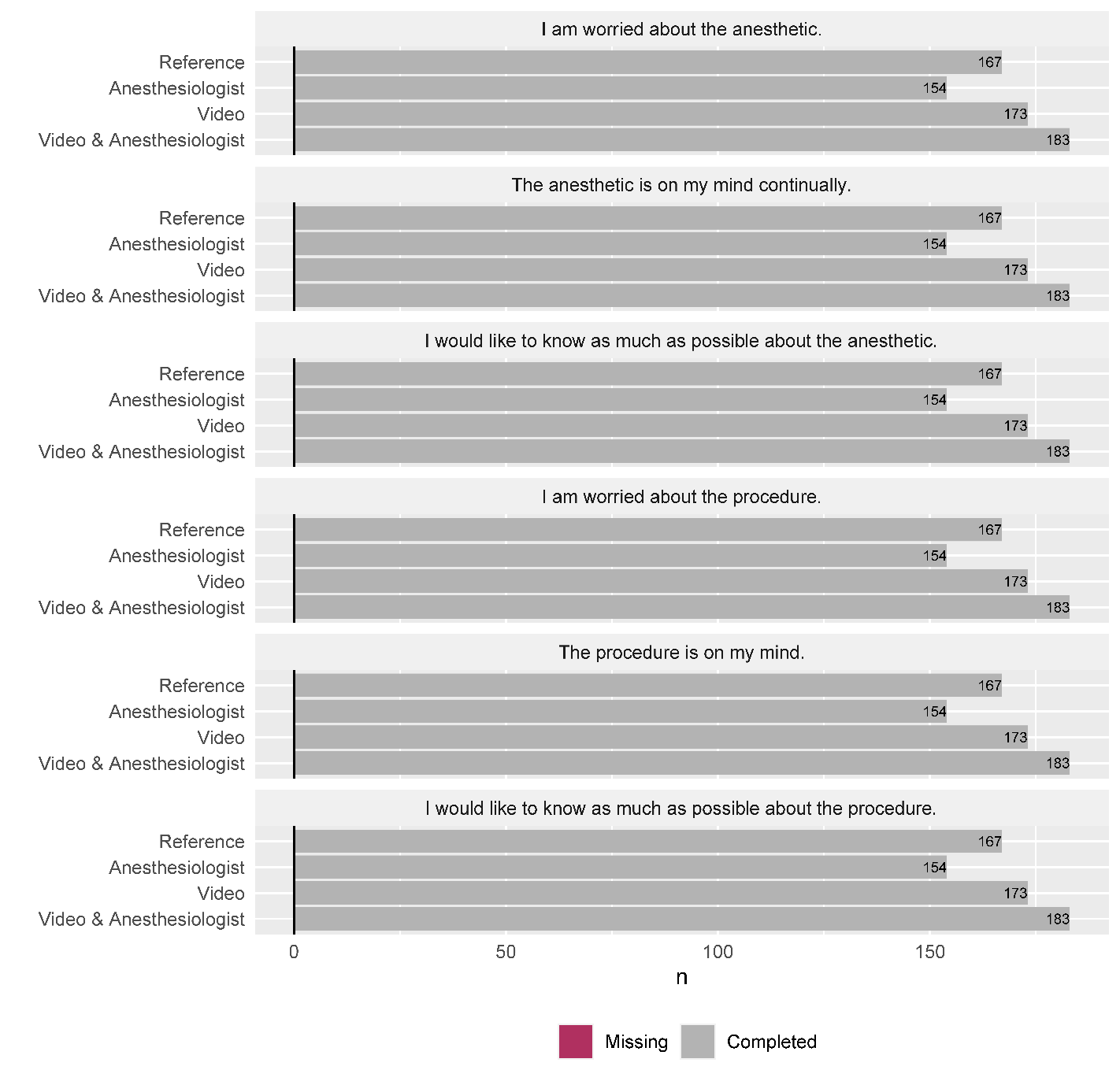 |

| **Supplemental Digital Content – Figure 2**  **Satisfaction and Subjective knowledge level vs. RAKQ scores** |
| --- |
|  |
| **Satisfaction** |
| 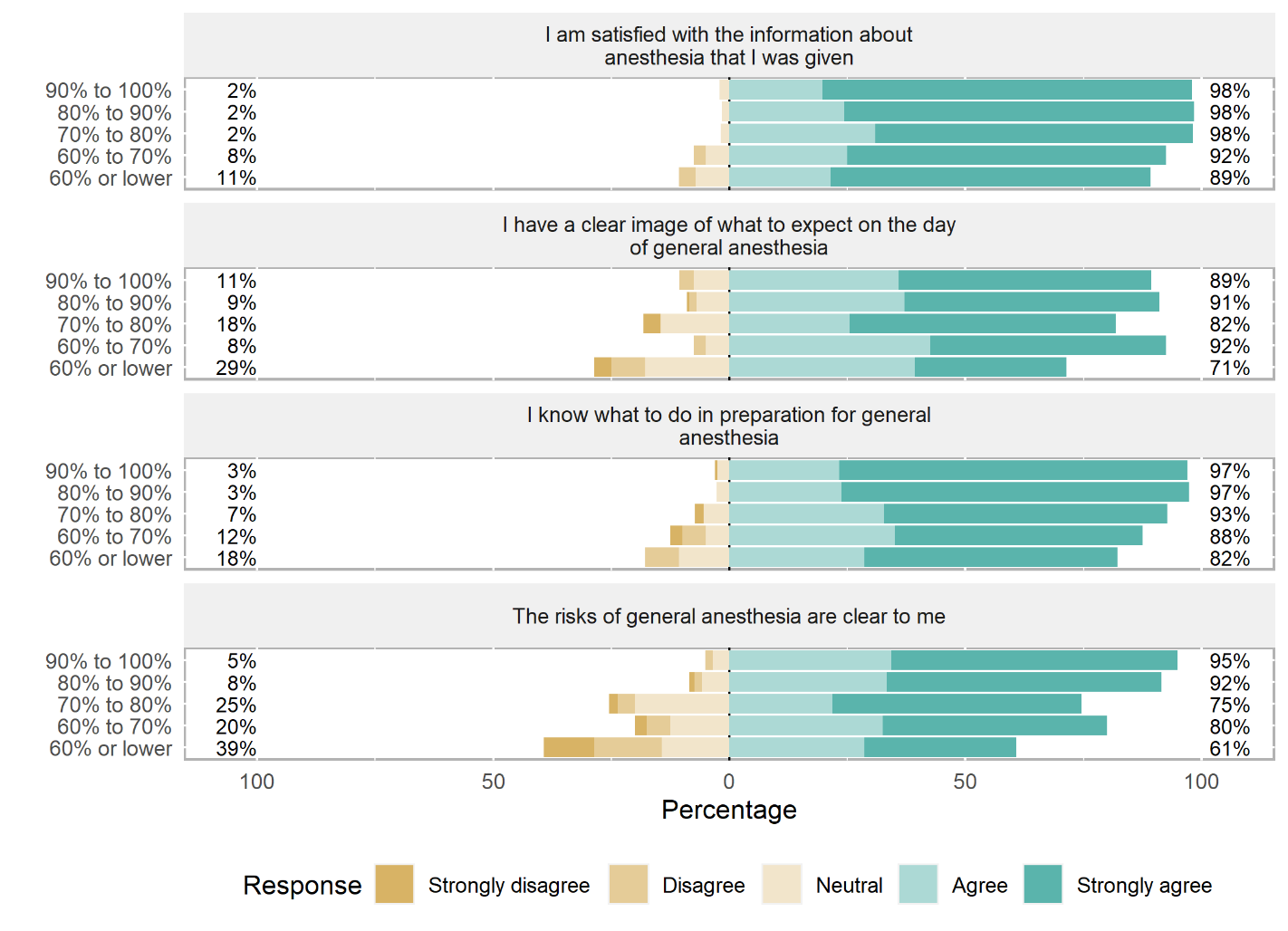 |
|  |
| **Subjective knowledge level** |
| 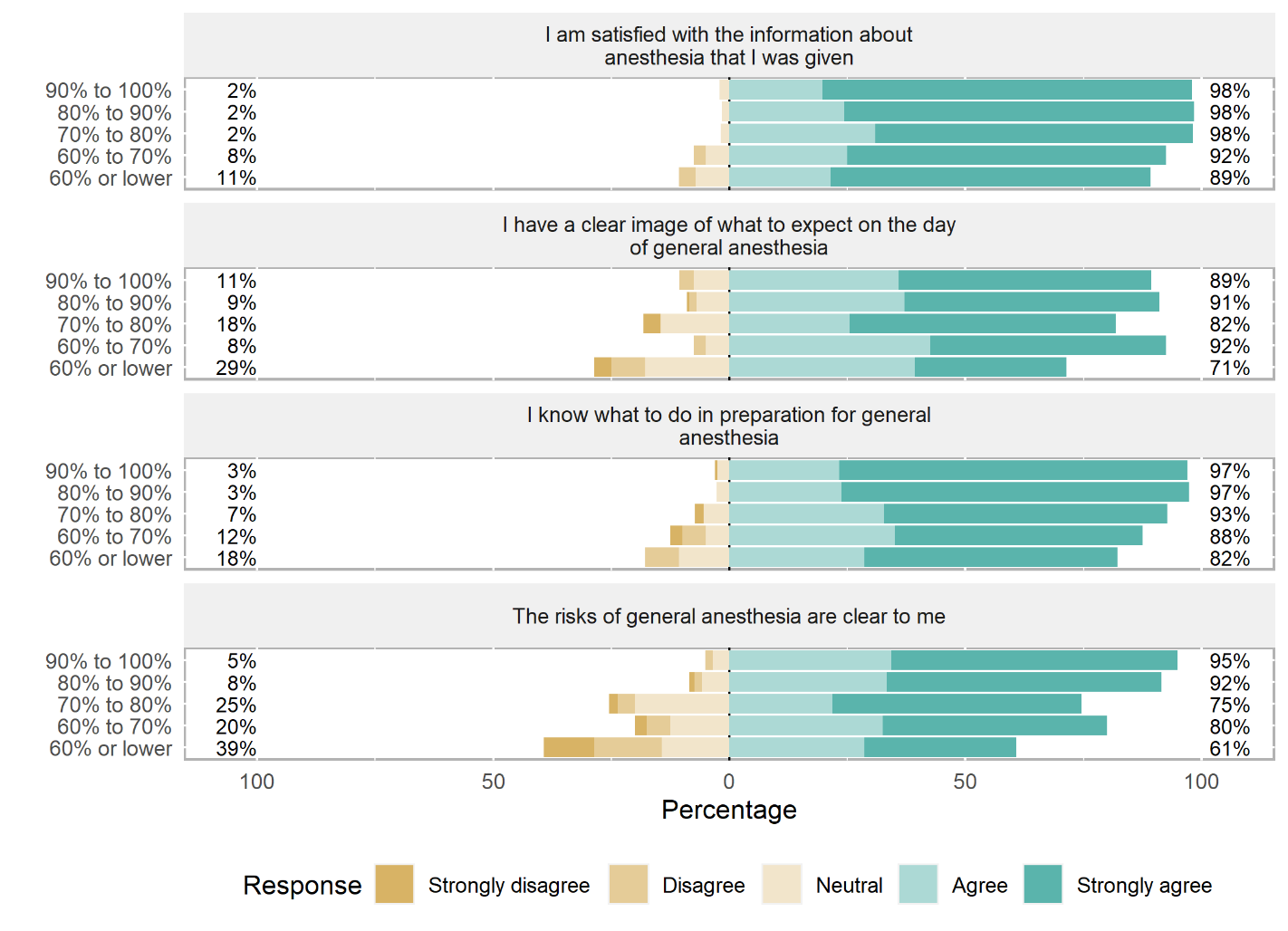 |
|  |
| **Distribution** |
| 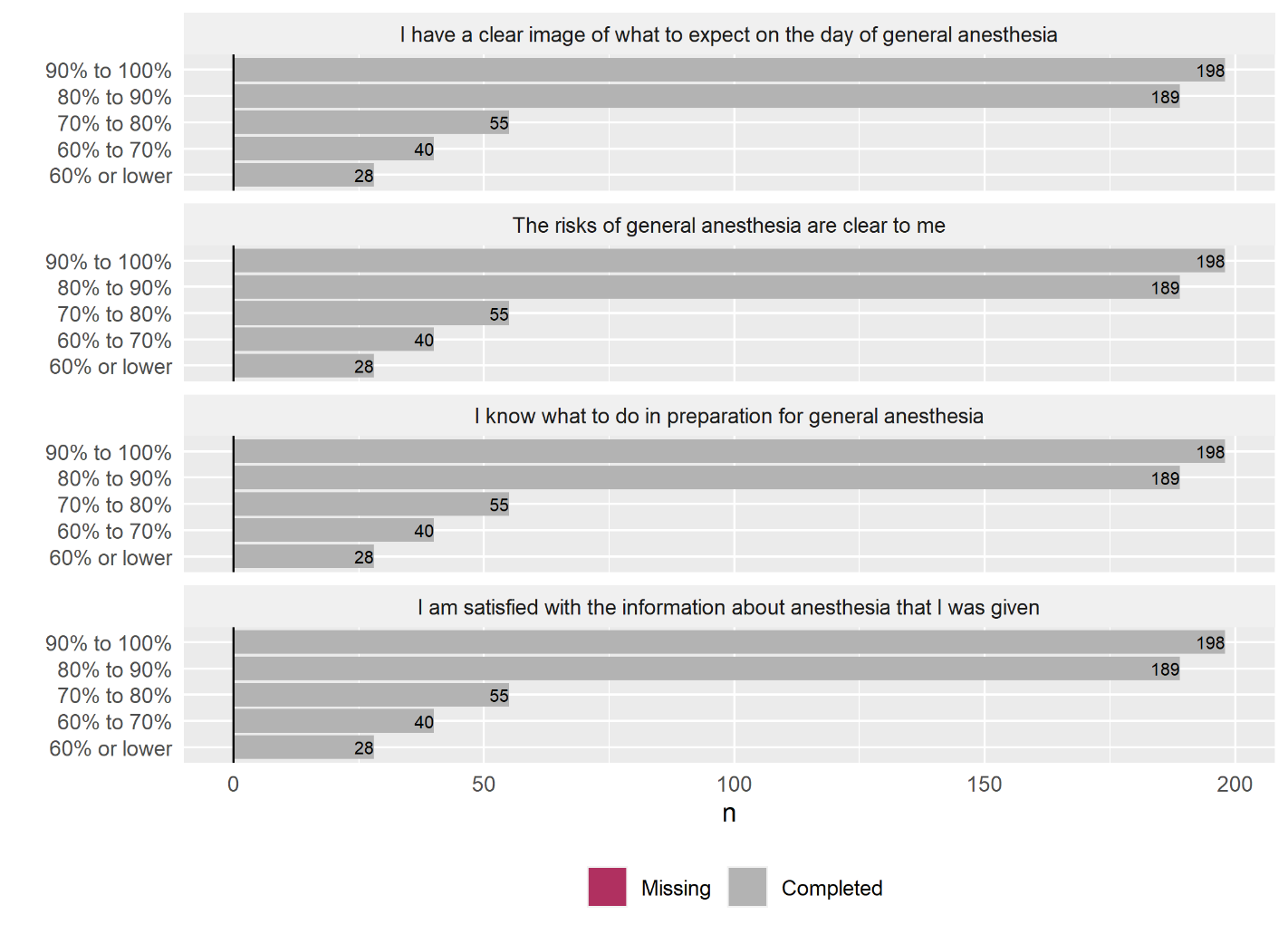 |
|  |
| Objective knowledge levels were categorised into five groups based on the percentage of correct answers on the Rotterdam Anaesthesia Knowledge Questionnaire (RAKQ). |
