# Supplementary material for: The effect of stand-alone and additional preoperative video education on patients’ knowledge of anaesthesia: A randomised controlled trial
Source: Eur J Anaesthesiol. 2024 Dec 19;42(4):313–23. doi: 10.1097/EJA.0000000000002109 (PMC11872255; doi:10.1097/EJA.0000000000002109)
Supplement: Supplemental Digital Content [file ejanet-42-313-s002.pdf]

Appendix F. Satisfaction and Subjective knowledge level vs. RAKQ scores

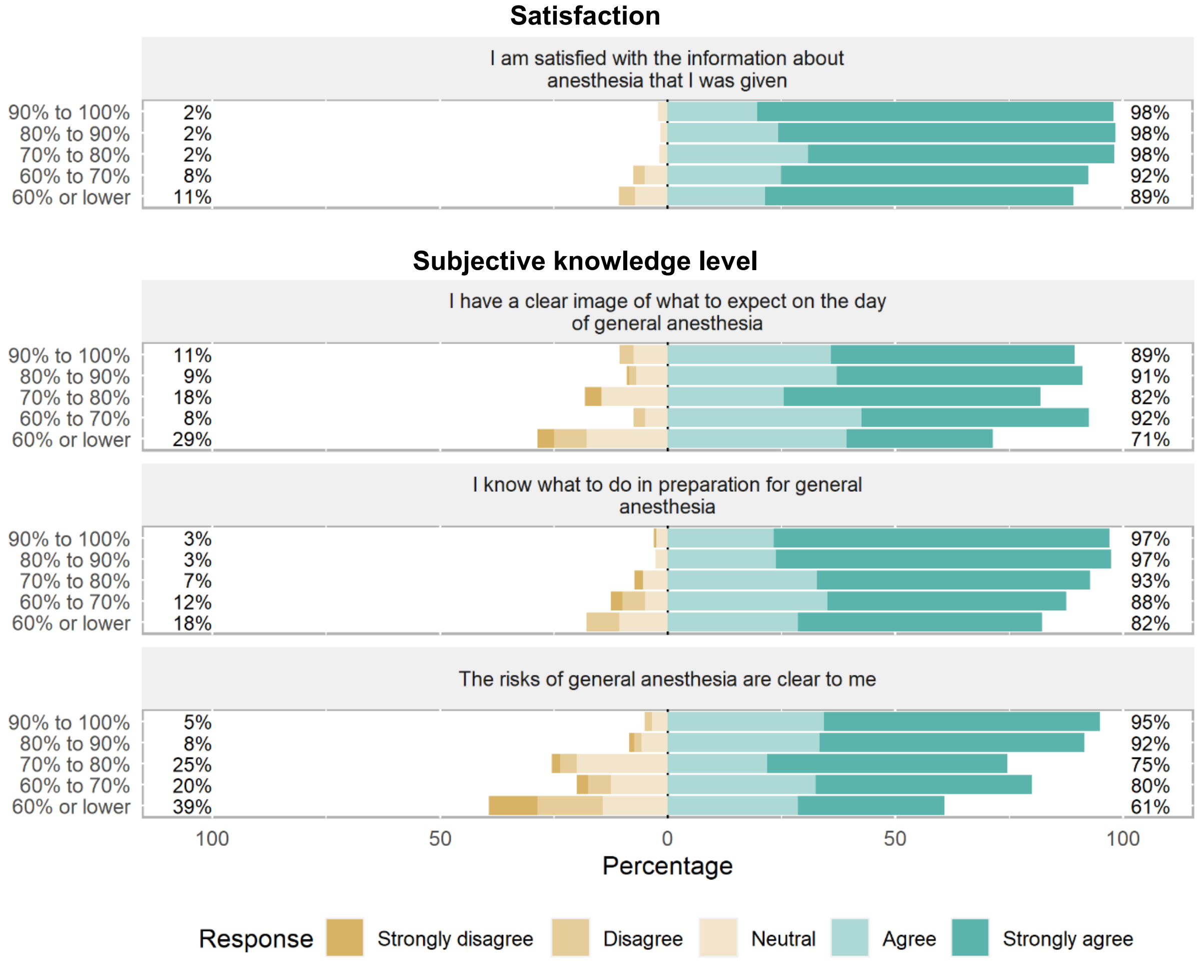

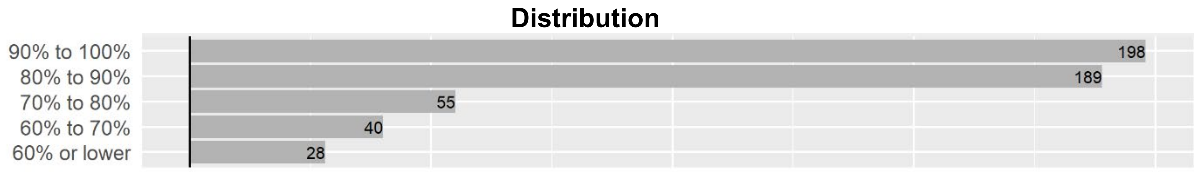

Objective knowledge levels were categorized in five groups based on the percentage correct answers on the RAKQ.
